# Supplementary material for: Integrated Transcriptomics and Metabolomics Analysis Reveals Convergent and Divergent Key Molecular Networks of Dominant Genic Male Sterility and Cytoplasmic Male Sterility in Cabbage
Source: Int J Mol Sci. 2025 Jan 31;26(3):1259. doi: 10.3390/ijms26031259 (PMC11818720; doi:10.3390/ijms26031259)
Supplement: Supplementary file 1 [file ijms-26-01259-s001.zip › supplementary figure.pdf]

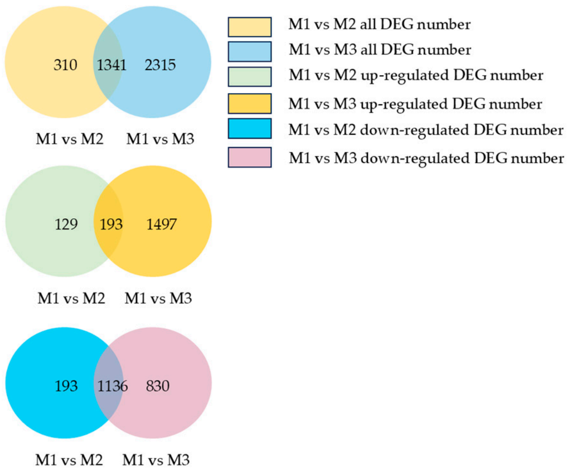

Figure S1: Venn analysis

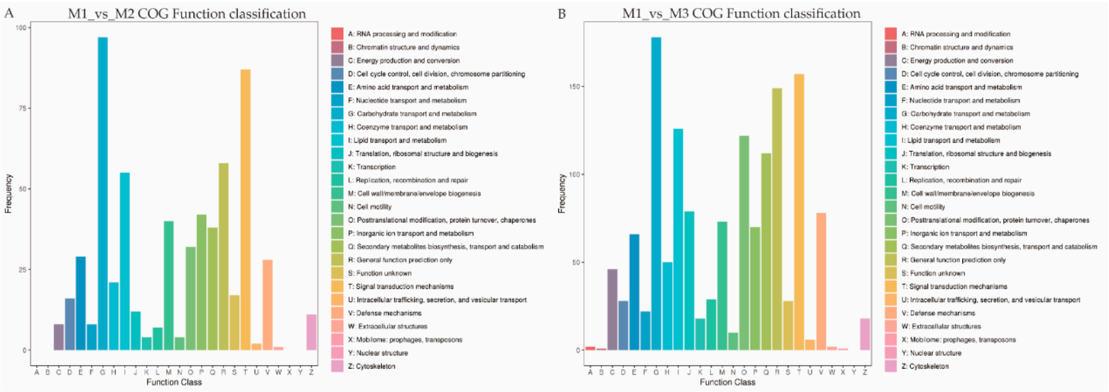

Figure S2 : Differently expressed genes COG annotation categorization statistic graph, horizontal coordinate is the content of each COG categorization, vertical coordinate is the number of genes.

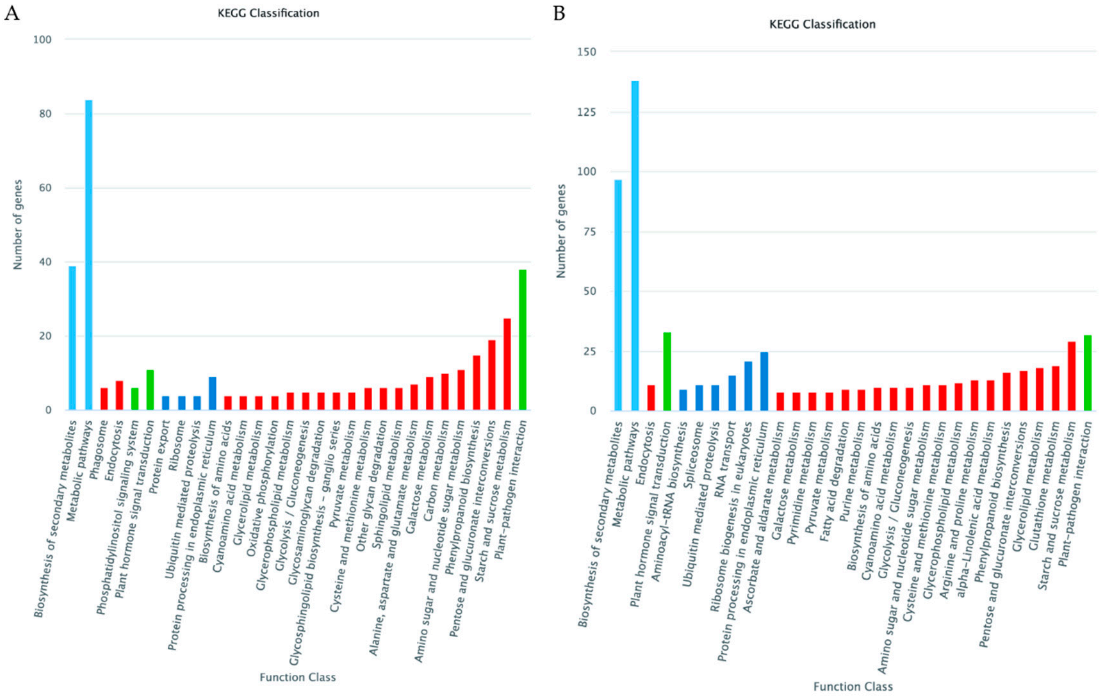

Figure S3. (A) KEGG maps of genes differentially and significantly expressed in both M1_vs_M2 and M1_vs_M3. (B) KEGG maps of genes differentially and significantly expressed only in both M1_vs_M3.

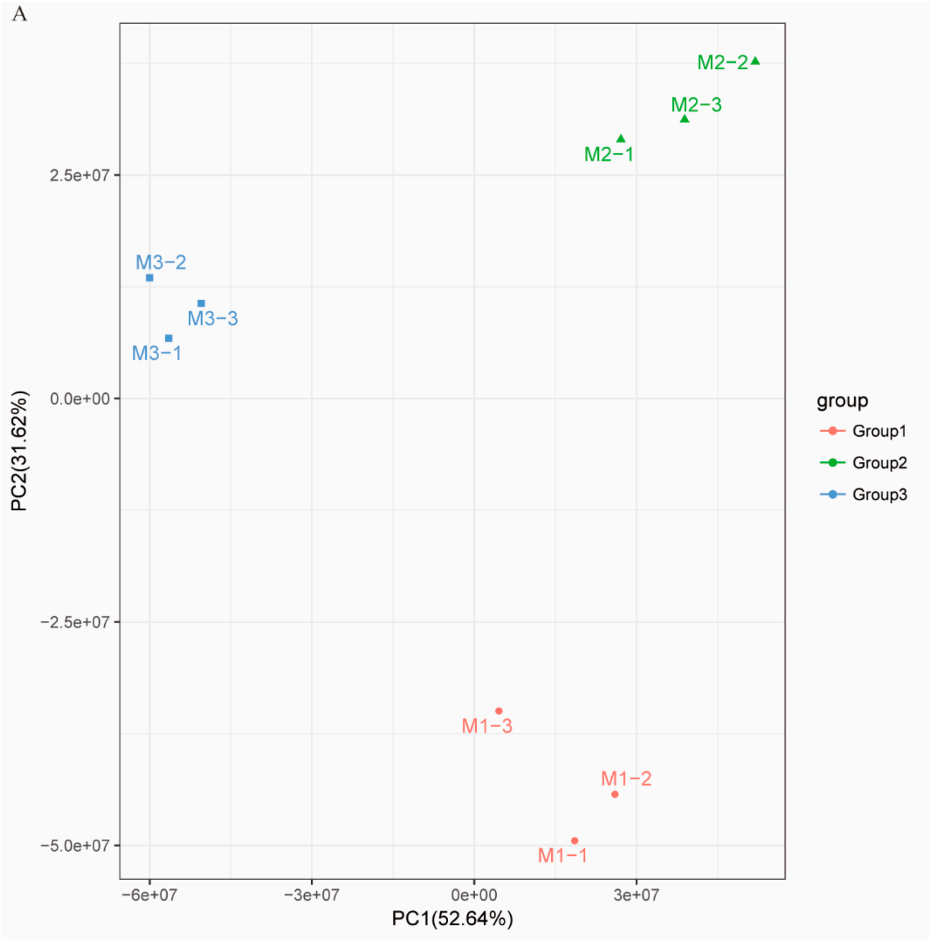

Figure S4: PCA analysis of all samples, where the X-axis denotes the first principal component and the Y-axis denotes the second principal component.

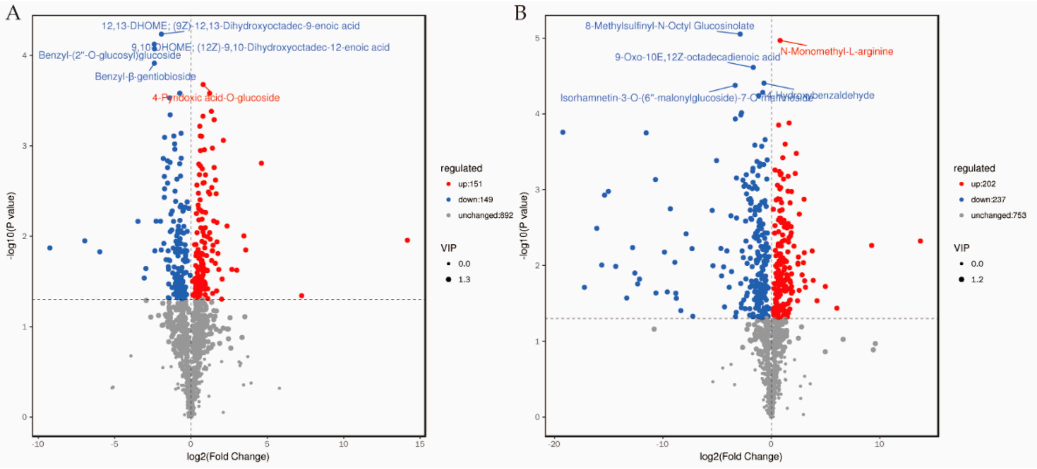

Figure S5: 代谢组 volcano

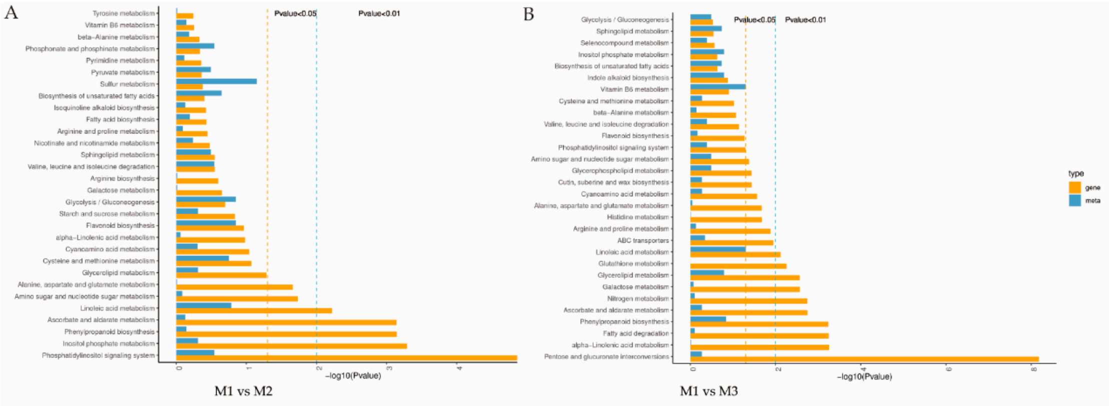

Figure S6: Top 30 pathways significantly enriched in differential genes/metabolites KEGG enrichment histograms. (A) M1_vs_M2; (B)M1_vs_M3
